# THE IMPACT OF IMMERSIVE VIRTUAL REALITY MEDITATION ON MENTAL HEALTH OF PEOPLE LIVING WITH DEMENTIA

**DOI:** 10.1093/geroni/igae098.3558

**Published:** 2024-12-31

**Authors:** Yongseop Kim, Jungjoo Lee, Dylan Kim, Junhyoung Kim

**Affiliations:** University of Utah, Salt Lake City, Utah, United States; University of Southern Mississippi, Hattiesburg, Mississippi, United States; Choate Rosemary Hall, Wallingford, Connecticut, United States; Texas A&M University, College Station, Texas, United States

## Abstract

Non-immersive, technology-based mindfulness meditation programs have been shown to effectively reduce depressive symptoms and improve the emotional health of older adults. Still, little research has been conducted to assess the mental health benefits of an immersive virtual reality meditation (IVRM) program among persons living with dementia (PLWD). The present study investigates the impact of the IVRM on depression, emotional health, and quality of life among PLWD. This study is based on a single-arm design. PLWD (n=8) received six sessions (three times a week for 2 weeks) of an IVRM program lasting 30-40 minutes each. The IVRM program is used as an individualized, customized meditation tool that provides more than 300 audio tracks, meditation process monitoring, and a selection of meditation environments. The findings indicated significant changes between pre and post interventions, showing a decrease in depression (M = 2.25, SD = 2.12; t(7) = 3.0, p <.05, d = 1.06) and an increase in emotional health (M = -3.87, SD = 4.05; t(7) = -2.71, p <.05, d = -0.95). However, a significant difference was not found in quality of life. This study provides suggestive evidence that the IVRM program led to the improvement in emotional health and a reduction in depression among PLWD. Healthcare professionals need to become more aware of the impact of the use of individualized VR-based meditation programs and learn how they might be integrated into their services to improve the emotional health and well-being of their clients.

